# Decline in air pollution and change in prevalence in respiratory symptoms and chronic obstructive pulmonary disease in elderly women

**DOI:** 10.1186/1465-9921-11-113

**Published:** 2010-08-22

**Authors:** Tamara Schikowski, Ulrich Ranft, Dorothee Sugiri, Andrea Vierkötter, Thomas Brüning, Volker Harth, Ursula Krämer

**Affiliations:** 1 Department of Epidemiology Institut für Umweltmedizinische Forschung (IUF) at the Heinrich-Heine-University Düsseldorf, Düsseldorf, Germany; 2 Chronic Disease Epidemiology Unit, Swiss Tropical and Public Health Institute, Associated Institute of the University of Basel, Basel, Switzerland; 3 University of Basel, Basel, Switzerland; 4 Institute for Prevention and Occupational Medicine of the German Social Accident Insurance (IPA), Ruhr-University Bochum, Germany

## Abstract

**Background:**

While adverse effects of exposure to air pollutants on respiratory health are well studied, little is known about the effect of a reduction in air pollutants on chronic respiratory symptoms and diseases. We investigated whether different declines in air pollution levels in industrialised and rural areas in Germany were associated with changes in respiratory health over a period of about 20 years.

**Methods:**

We used data from the SALIA cohort study in Germany (*S*tudy on the influence of *A*ir pollution on *L*ung function, *I*nflammation and *A*ging) to assess the association between the prevalence of chronic obstructive pulmonary disease (COPD) and chronic respiratory symptoms and the decline in air pollution exposure. In 1985-1994, 4874 women aged 55-years took part in the baseline investigation. Of these, 2116 participated in a questionnaire follow-up in 2006 and in a subgroup of 402 women lung function was tested in 2008-2009. Generalized estimating equation (GEE) models were used to estimate the effect of a reduction in air pollution on respiratory symptoms and diseases.

**Results:**

Ambient air concentrations of particulate matter with aerodynamic size < 10 μm (PM_10_) declined in average by 20 μg/m^3^. Prevalence of chronic cough with phlegm production and mild COPD at baseline investigation compared to follow-up was 9.5% vs. 13.3% and 8.6% vs. 18.2%, respectively. A steeper decline of PM_10 _was observed in the industrialized areas in comparison to the rural area, this was associated with a weaker increase in prevalence of respiratory symptoms and COPD. Among women who never smoked, the prevalence of chronic cough with phlegm and mild COPD was estimated at 21.4% and 39.5%, respectively, if no air pollution reduction was assumed, and at 13.3% and 17.5%, respectively, if air pollution reduction was assumed.

**Conclusion:**

We concluded that parallel to the decline of ambient air pollution over the last 20 years in the Ruhr area the age-related increase in chronic respiratory diseases and symptoms appears to attenuate in the population of elderly women.

## Introduction

Several epidemiological studies have shown that chronic exposure to high levels of air pollutants (PM_10 _and NO_2_) has adverse effects on respiratory health. These adverse effects on respiratory health are not limited to high concentrations of air pollutants, but have also been observed at relatively low concentrations. It has been previously reported that long-term exposure to air pollutants from traffic related sources reduce lung function [1-5] and influence chronic respiratory diseases [6-8]. Furthermore, long-term exposure to air pollutants is known to be associated with cardiovascular mortality [9-12] and increased hospital admissions [13-16].

However, less is known about the effect of a reduction in air pollutants on chronic respiratory symptoms and diseases, including chronic cough. Chronic cough is common in people aged 70 and over and the prevalence increases further with age [17-21]. Additionally, chronic cough may also be the first symptom in the development of chronic obstructive pulmonary disease [21,22].

There is evidence that a reduction in air pollutants attenuates the decline in respiratory health in children. The delay in lung function development, due to air pollutants, attenuates when the children move to cleaner areas [23,24]. Moreover, a recent prospective cohort study of adults living in Switzerland, the *S*wiss study on *A*ir *P*ollution and *L*ung *D*isease in *A*dults (SAPALDIA), showed that a decline in lung function [25], as well as an increase of respiratory symptoms [26], is attenuated by a reduction in exposure to PM_10_. However, the effect of a reduction in air pollutants on respiratory health in elderly people has not been analysed so far.

In the present study, we investigated whether the age-related increase of respiratory symptoms and diseases is attenuated by a reduction in exposure to ambient air pollutants using data collected from the SALIA study (the *S*tudy on the influence of *A*ir pollution on *L*ung function, *I*nflammation and *A*ging), a prospective cohort of elderly women living in the highly industrialised Ruhr district and in adjacent rural areas in Germany. Chronic respiratory symptoms and lung function were first measured in 1985-1994, when ambient air pollution exposure was high, and follow-up was conducted from 2006 to 2009, when concentrations of ambient air pollutants in the Ruhr district had been considerably reduced. Thus, we were provided with sufficient power to examine whether the changes in prevalence of respiratory symptoms and disease were attenuated by reduction in ambient air pollutants.

## Materials and methods

### Design and study population

**Figure 1:**
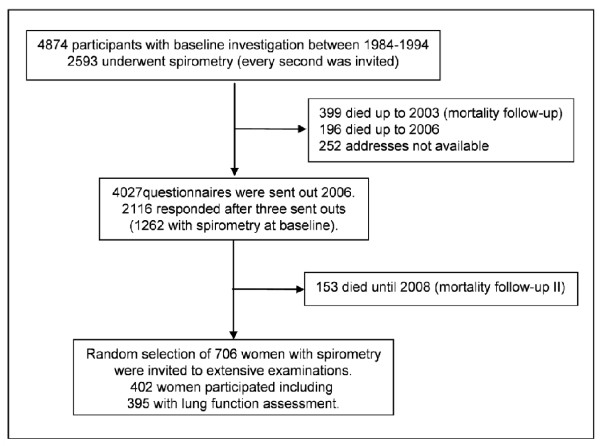
**Flowchart showing the SALIA collective from baseline till follow-up in 2007/2008**.

The SALIA study was initiated in the early 1980 s by the North Rhine-Westphalia State Government to investigate the effect of air pollution exposure in women. The study population was a sample of women from the Ruhr area, Germany, and two rural areas in the North of the Ruhr area. Health examinations were conducted between 1985 and 1994 in 4874 women, who were all approximately 55-years of age at the time of examination. Health examinations included lung function measurements for a subset of the participants (n = 2,593). Previous results of the baseline investigation showed that exposure to high concentrations of air pollutants reduces lung function and was associated with COPD [6,10]. In 2006, a follow-up study of the same women was conducted to assess the changes in respiratory symptoms and diseases in these women after a strong decline in concentrations of ambient air pollutants in the Ruhr area. A questionnaire about respiratory health and its risk factors was sent out to all surviving participants, each of whom received three reminder letters. Completed questionnaires were received from 2116 (53%) of the surviving participants. In 2007 to 2009 a follow-up examination in a subgroup of the study population was conducted. This subgroup consisted of 706 women who had a lung function measurement at baseline and who agreed to further examinations in the questionnaire follow-up in 2006. The women were invited in a randomized manner from four cities in the Ruhr area (Duisburg, Dortmund, Essen and Gelsenkirchen), as well as the rural county of Borken, which was used as a reference area. In total, 402 women, who were aged 70 to 80 years old, participated and lung function testing was completed in 395 of these participants. Figure 1 gives a flow chart of the SALIA cohort study between baseline investigation and follow-up. The present analysis was restricted to the women who had complete information on respiratory health outcomes at baseline investigation and at the follow-up. Approval of the study was obtained from the Ethical Committee of the University of Bochum. We received written informed consent from all participants.

### Assessment of respiratory health and risk factors by questionnaire

Together with an invitation to participate in the baseline investigation, the women received a self-administered questionnaire about respiratory health and its risk factors. The same questions regarding respiratory symptoms and diseases of the baseline investigation were used in the follow-up. We asked whether a physician had ever diagnosed chronic bronchitis and additionally we asked for respiratory symptoms. Respiratory symptoms were divided in two categories: "frequent cough in the morning or during the day a) without phlegm production or b) with phlegm production". We additionally collected information about the following known risk factors for respiratory diseases in the questionnaire: current and past smoking habits, passive smoking exposure at home or at work, indoor exposure by heating with fossil fuels, and occupational exposures to dust or fumes. For smoking habits, the women were grouped as never smoker, passive smoker (at home or/and at work place), past smoker or current smoker. We classified socioeconomic status at baseline into four categories using the highest school level achieved by either the women or her husbands as low (< 10 years), medium (= 10 years) or medium high (11-12 years) and high >12 years).

### Lung function measurement and COPD definition

Spirometry was performed according to the ATS/ETS recommendations [25]. Forced expiratory volume in 1 second (FEV_1_) and forced vital capacity (FVC) were measured. Between three to four manoeuvres were performed under direction of trained personnel, and the values where the maximal FEV_1 _was reached were used. All measuring instruments were calibrated prior to each testing. The technical personnel were trained and all results were reviewed by a pulmonary physician. COPD was defined using the ratio FEV_1_/FVC, which is considered a sensitive measure of COPD on its own [26]. We defined two forms of COPD: mild COPD (stage 1) was defined as FEV_1_/FVC ratio < 0.7 and the moderate form (stage 2) as FEV_1_/FVC ratio <0.7 and FEV_1 _< 80% of predicted value. Both constitute the main criterion for COPD according to the Global Initiative for Chronic Obstructive Lung Disease (GOLD) criteria [27]. However, we used a modified version of the GOLD criteria as we did not use post-bronchodilator measurements in our analysis. We therefore excluded women who reported asthma from the analysis, in which COPD was the outcome, to avoid confounding. Asthma was considered present when ever diagnosed by a physician (Table 1). We additionally conducted a sensitivity analysis including women with asthma.

**Table 1:** Characteristics of the study population of elderly women at baseline and at follow-up

	Baseline 1985-1994		Follow-up 2006		Follow-up 2007/2008	
	**N**	**Mean ±SD**	**N**	**Mean ±SD**	**N**	**Mean ±SD**

**Age (year)**	2115	54.5 ± 0.7	2106	71.3 ± 3.3	402	74.1 ± 2.6
**BMI (kg/m^2^)**	1874	27.2 ± 4.2	2048	27.0 ± 4.7	402	27.6 ± 4.6

	**N**	**%**	**N**	**%**	**N**	**%**

**Urban residency ^a^**	2116	55.7	2116	55.7	402	52.7
**Smoking status:**						
**Passive smoking**	2091	47.1	2014	14.5	402	5.7
**Ex smoker**	1758	9.0	2063	14.6	402	15.7
**Current smoker**	2112	11.9	2063	5.6	402	3.0
**Indoor exposure ^b^**	2081	18.2	1809	12.6	402	12.9
**Education:**	2098	-	2098	-	402	-
**< 10 years**	-	22.0	-	22.0	-	17.3
**= 10 years**	-	48.9	-	48.9	-	51.0
**11-12 years**	-	18.1	-	18.1	-	19.7
**> 12 years**	-	11.0	-	11.0	-	12.2
**Asthma**	2074	2.0	2021	5.4	401	9.7
**Hypertension**	2077	23.3	1997	53.4	400	66.3

SD: Standard deviation

BMI: Body mass index (weight/height^2^)

^a ^Urban. Duisburg, Dortmund, Essen, Gelsenkirchen; rural: Borken

^b ^Heating with fossil fuels (gas, coal or wood)

### Air pollution measurements

In the assessment of air pollution, we used data from local monitoring stations maintained by the State Environmental Agency of North Rhine-Westphalia since more than 25 years. These monitoring stations are designed to reflect broad scale spatial variations in air quality. All monitoring stations used in this study were located within a distance of not more than 8 km to the women's home address. The individual exposure to background ambient air pollution at baseline and follow-up investigation was estimated by the PM_10 _and NO_2 _concentrations of the monitoring station located nearest to the participant's residential address. To assess long-term exposure, we used the 5-year mean concentrations of PM_10 _and NO_2_. For characterizing long-term exposure at baseline, we used the five year mean of the year of the baseline examination (within 1985 to 1994) and the preceding four years and, for exposure at follow-up, the means of the years 2002 to 2006. Due to the incompleteness of air pollution data from Borken, where continuous measurements only started in 1990, monitoring data proceeding this year were imputed by using measurements from 1981 to 2000 from 15 monitoring stations in the Ruhr area assuming similar trends. The imputation was performed by using linear regression modelling with air pollution as the depended variable, year of measurement as the independent variable and an autoregressive correlation between repeated measurements performed at the same measurement site using air pollution measurements from 1981 to 2000 [10]. Between 1985 and 1987, discontinuous measurements were performed in Borken (four days per month), and these agreed well with the imputed values [6].

### Statistical analysis

The association between air pollution levels and the prevalence, and changes in prevalence, of COPD and respiratory symptoms at baseline and at follow-up was analyzed using generalised estimating equations (GEE). The individual change in exposure *ΔE *was calculated as the difference between the baseline measurement and the measurement at follow-up. For multivariate regression modelling, we assumed linear dependency of the prevalence of chronic respiratory diseases and symptoms on exposure at baseline (*E_baseline_*), and on the time of follow-up *t*, since all women were 55 years of age at baseline. Additionally, we investigated whether the age related increase in diseases or symptoms associated with the time of follow-up *t *was linearly modified by the change in exposure *ΔE*.

The GEE model controlled for a set of potential confounder (smoking behaviour, passive smoking, social status and exposure to indoor air pollutants) on an individual basis. However inclusion of social status, indicated by school education, passive smoking and indoor air pollution exposure did not change the parameter estimates by more than 10% and were not included in the final model.

The final models were written as follows:

$$\begin{array}{l} p_{0}=\beta_{0}+\beta_{1}*E_{baseline}+\beta_{4}*S_{baseline}\text{ and}\\ p_{t}=\beta_{0}+\beta_{1}*E_{baseline}+(\beta_{2}+\beta_{3}*\Delta E)*t+\beta_{4}*S_{baseline}+\beta_{5}*S_{follow-up} \end{array}$$

with: *p_0 _*prevalence at baseline, *p_t _*prevalence at follow-up, *E_baseline _*exposure at baseline, *ΔE *exposure decline (exposure at baseline minus exposure at follow-up), t follow-up time, *S_baseline _*smoking at baseline (yes = 1, no = 0) and *S_follow-up _*smoking at follow-up (yes = 1, no = 0).

All statistical analyses were performed with SAS for windows release 9.1 (SAS Institute, Cary, NC).

## Results

### Characteristics of study participants

The characteristics of the study cohort are presented in Table 1. The majority of the study participants lived in cities of the Ruhr area. The mean age of these women at the follow-up investigation in 2006 was 71.3 years. Little change in body mass index (BMI) was observed. Most women tended to give up smoking; similarly, passive smoke exposure was considerably reduced between baseline and follow-up investigation. A reduction of heating with fossil fuels was reported throughout the areas. The majority of women had a school education of 10 or more years. A slight increase in reported asthma and a doubling of reported hypertension was observed between the baseline investigation and the follow-up. Twelve per cent of the women were occupationally exposed to dust and fumes before baseline investigation, but not afterwards. Occupational exposure to dust and fumes was not considered as a potential risk factor in the proceeding analysis. More than 98% of the participants had not moved since baseline. We also evaluated whether participants from the 2006 survey differed from non-participants. Length of education was a primary differentiating factor; less than 10 years of schooling was reported by 21.6% of those responding at baseline, by 38.5% of those 595 women who died between baseline and follow up, and by 36.6% of those 1911 not responding but who were still alive. We additionally examined whether the associations between air pollution and respiratory health as reported in a previous publication of the same cohort [28] differed between the responder groups, but did not detect any systematic differences; no significant interactions were observed between responder status and air pollution on respiratory health.

### Prevalence of respiratory health outcomes

The prevalence of chronic bronchitis, respiratory symptoms and COPD are shown in Table 2. The prevalence of respiratory symptoms and chronic bronchitis by physician's diagnosis increased between the baseline investigation and 2006 with increasing age of the participants. Participants who had missing answers in the questionnaire were excluded from the analysis, so the numbers vary slightly from one respiratory health outcome to another. Chronic cough was the most commonly reported respiratory symptom with a prevalence of 20.6% and 26.5% at baseline and at follow-up, respectively. The prevalence of mild COPD assessed with FEV_1_/FVC < 0.7 at baseline was 8.6% and 18.2% at follow-up and, therefore, comparable to the prevalence of chronic cough with phlegm production. Only a few participants were classified as having moderate COPD (n = 14 and n = 23, respectively). At the baseline investigation the prevalence of all respiratory symptoms and diseases was lower in the rural than in the urban areas whereas this was not true for the follow-up investigation.

**Table 2:** Prevalence of respiratory symptoms, chronic bronchitis at baseline (1985 - 1994) and at follow-up (2006) and COPD at baseline (1985-1994) and at follow-up (2007/2008) in a subgroup of elderly women

	Prevalence					
	Baseline			Follow-up		
**Respiratory health outcome**	**all**	**Ruhr area**	**rural areas**	**all**	**Ruhr area**	**rural areas**


**Chronic Bronchitis by physician's diagnosis ^a ^**	N = 2073 (8.2%)	N = 1167 (9.3%)	N = 905 (6.6%)	N = 2032 (11.6%)	N = 1125 (13.6%)	N = 90782 (9.0%)
**Chronic cough ^a ^**	N = 2110 (20.6%)	N = 1175 (22.7%)	N = 935 (18.0%)	N = 1947 (26.5)	N = 1079 (27.9%)	N = 868 (24.7%)
**Chronic cough with phlegm production ^a^**	N = 2099 (9.5%)	N = 1168 10.1%	N = 931 8,8%	N = 1979 (13.3%)	N = 1098 (13.5%)	N = 890 (13.2%)
						
**Mild COPD^b ^FEV_1_/FVC < 0.7**	N = 384 (8.6%)	N = 201 10.5%	N = 183 6.6%	N = 347 (18.2%)	N = 179 (12.9%)	N = 163 (22.7%)
**Moderate COPD^b^FEV_1_/FVC< 0.7 and FEV_1_< 80% predicted**	N = 384 (3.7%)	N = 201 5.0%	N = 183 2.2%	N = 347 (6.6%)	N = 179 (5.6%)	N = 163 (8.0%)

^a^Reported by participant

^b^Only in a subgroup that was invited for the follow-up examination in 2007/2008. Women with asthma were excluded

### Change in concentrations of PM_10 _and NO_2_

**Figure 2:**
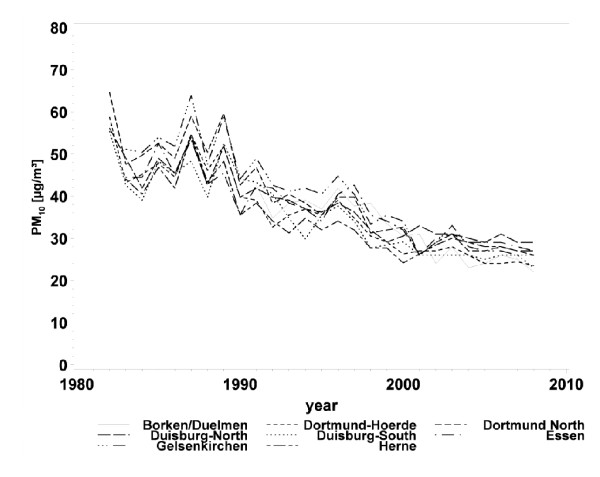
**Annual mean concentrations of particulate matter with a diameter of less than 10 μm (PM_10_)**.

**Figure 3:**
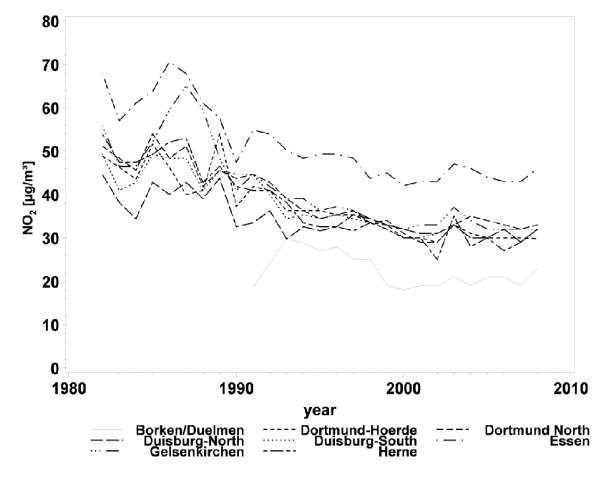
**Annual mean concentrations of nitrogen dioxide (NO_2_) from 1982 to 2008 by study area**.

A strong decrease in air pollution levels was observed throughout the entire study area (Table 3). In particular, urban areas with high PM_10 _levels at baseline experienced a strong reduction in concentrations through to follow-up (Figure 2). Across the 5 study areas, the 5-year mean PM_10 _concentrations declined on average from 46.6 μg to 26.9 μg (interquartile range: 10 μg/m^3^). A slightly weaker decline was observed for NO_2 _concentrations (Figure 3). In the rural area of Borken, NO_2 _concentrations remained stable during the 20 years of the follow-up, but the 5-year mean concentrations of NO_2 _decreased in average from 38.1 μg to 27.9 μg (interquartile range: 12.2 μg/m^3^).

**Table 3:** Distribution of long-term air pollution exposures among women living in the Ruhr area and an adjacent rural area in Germany at baseline and at follow-up

	Total Group (n = 2116)							
	**Baseline in** **1985-1994**		**Follow-up in** **2006**		**Change in** **rural area**		**Change in urban area**	

	**PM_10_**	**No_2_**	**PM_10_**	**NO_2_**	**PM_10_**	**NO_2_**	**PM_10_**	**NO_2_**

**Min**	38.9	22.0	25.0	20.2	13.9	1.8	14.8	8.6
**25 Percentile**	42.6	24.4	25.0	20.2	14.3	2.6	16.2	13.4
**Median**	46.9	39.8	26.0	31.2	14.3	4.2	23.1	16.4
**Mean**	46.6	38.1	26.9	27.9	17.6	3.8	21.4	15.4
**75 Percentile**	52.1	49.8	28.4	32.8	21.9	4.8	24.6	17.2
**Max**	53.6	61.0	30. 5	44.6	24.0	6.3	25.2	21.2

### Decline in air pollution exposure and change of prevalence of respiratory health outcomes

The association of decline in PM_10 _and NO_2 _pollution between baseline and follow-up with the prevalence of respiratory symptoms and diseases at baseline and at follow-up is presented in Table 4. The table shows the mutually adjusted parameter estimates (prevalence change per unit) for smoking at baseline, change in smoking behaviour between baseline and follow-up, baseline exposure, follow-up time and finally for the change in exposure between baseline and follow-up.

**Table 4:** Results of GEE regression analysis: Association of prevalence of chronic bronchitis, respiratory symptoms, and COPD with smoking at baseline and at follow up, exposure at baseline, follow-up time and exposure decline; coefficients are mutually adjusted

	Chronic bronchitis ^a^		Chronic cough		Chronic cough with phlegm production		Mild COPD ^b^		Moderate COPD ^c^	
	PM_10_	NO_2_	PM_10_	NO_2_	PM_10_	NO_2_	PM_10_	NO_2_	PM_10_	NO_2_
**Sample size**	1950		1902		1922		342		342	

	**Parameter estimates and 95% confidence interval (times 100)**									

**Intercept**	-1.51-12.04;9.02	4.33 *0.94;7.73	0.24-14.85;15.33	15.05 *10.15;19.95	5.10-6.06;16.26	9.07 *5.48;12.67	-2.81-28.64;23.01	3.93-3.47;11.33	-15.52 *-24.83;-6.21	0.15-4.04;4.34
**Smoking at baseline**	2.42-1.31;6.16	1.95-1.66;5.57	13.94 *8.35;19.54	13.99 *8.36;19.62	5.92 *1.64;10.20	6.08 *1.77;10.39	3.92-5.34;13.19	4.67-4.95;14.28	7.02-0.89;14.92	7.62-1.01;16.24
**Smokingat follow-up**	-1.20 -7.45;5.06	-1.57-7.83;4.68;	14.59 *5.86;23.32	14.46 * 5.73;23.20	7.82 * 1.56;14.08	7.96 *1.83;14.09	-9.25-27.72;9.22	-9.45-27.38;8.48	--2.18-16.06;11.69	2.04-16.02;11.93
**Exposure at baseline ^d^**	2.01-0.25;4.26	2.32 *0.18;4.47	4.04 *0.80;7.28	2.69-0.40;5.77	0.82-1.56;3.20	-0.07-2.25;2.11	2.35-3.24;7.94	2.97-1.99;7.94	3.96 *1.60;6.32	2.05-0.96;5.06
**Follow up time ^e^**	2.22-2.24;6.69	1.82 *0.28;3.37	12.58 *5.80;19.36	5.37 *2.77;7.98	8.22 *3.07;13.37	3.60 *1.60;5.59	20.61 *7.81;33.41	9.12 *4.78;13.46	8.02 *0.01;16.03	2.73 *0.03;5.43
**Follow up time exposure decline ^f^**	-0.17-4.37;4.03	0.21-1.08;1.50	-8.17 *-14.54;-1.79	-1.15-3.25;0.96	-5.39 *-10.22;-0.57	-0.87-2.41;0.66	-14.62 *-25.88;-3.36	-4.64 *-8.03;-1.26	-6.20-13.33;0.94	-1.66-3.8;0.048

*p < 0.05

^a ^Reported physician's diagnosis

^b ^FEV1/FVC <0.7

^c ^FEV1/FVC <0.7 and FEV1 < 80% predicted

^d ^unit: PM_10 _10 μg/m^3^, NO_2 _25 μg/m^3^

^e ^unit: 10 yr

^f ^unit: PM_10 _20 μg/m^3 ^/10 yr, NO_2 _10 μg/m^3 ^/10 yr

The prevalence of respiratory health outcomes increased with increasing age of the cohort. Exposure to ambient air pollution at baseline was also an important risk factor for respiratory health. However, with the exception of chronic bronchitis, the increase in prevalence of cough, without and with phlegm production, as well as of mild and moderate COPD were significantly (p < 0.05) attenuated by the decline of background concentration of PM_10 _in ambient air (for moderate COPD p < 0.09). For an observed decline of NO_2 _background concentration in ambient air by approximately 10 μg/m^3^, the respective effect on the respiratory health outcomes was only marginal. Smoking at baseline was a strong risk factor for chronic cough with and without phlegm production, but quitting smoking between baseline and follow-up significantly reduced the prevalence of these respiratory symptoms. A decrease in PM_10 _by 20 μg/m^3 ^over a period of 10 years of follow-up attenuated the prevalence of the age-related increase of chronic cough with and without phlegm production, as well as mild COPD.

Industrialized and rural areas might differ in some respects, which we did not account for in our analysis. Therefore as a sensitivity analysis we repeated the analysis only including women from urban areas (data not shown). The parameter estimates for prevalence of cough, with and without phlegm production, were similar but less significant. Chronic bronchitis now showed a reduction in prevalence due to the decline in both the exposures of PM_10 _(p < 0.270) and in NO_2 _(p < 0.049). All other results remained unchanged.

In order to address whether potentially erroneous assessing of smoking might have affected the results, we additionally did all analysis only including non smoking women into the analysis. The parameter estimates varied in an unsystematic way. The effect for chronic cough was slightly stronger, whereas the effect for COPD was slightly weaker, the significance remained the same.

As a further sensitivity analysis we also repeated the analysis for COPD as defined by lung function without excluding women reporting asthma. All results were slightly stronger (data not shown) and significance remained.

Table 5 summarizes the comparison of the model estimated and observed prevalence of respiratory symptoms and COPD for participants who never smoked. We calculated estimated prevalences using the model equations given in paragraph 2.5 'Statistical analysis' and the results of GEE regression analysis given in table 4. Estimated and observed prevalence at baseline and at follow-up were very similar. Furthermore, the GEE model allowed for estimating the prevalence, if no exposure decline would have occurred, and the estimated prevalence of this counterfactual scenario demonstrated an attributable effect of air pollution decline. For an exposure decline of PM_10 _of 20 μg/m^3 ^within 15 years, a hypothetical attenuation of the prevalence of respiratory symptoms and COPD between 8% and 20% was estimated, respectively. Among, women who never smoked, the prevalence of chronic cough with phlegm production and mild COPD was estimated at 21.4% and 39.5%, respectively, if no ambient air PM_10 _reduction was assumed. However, these estimates were changed to 13.3% and 17.5%, respectively, if air pollution reduction as observed was assumed. For an exposure decline of NO_2 _of 10 μg/m^3^, the attributable effect was considerably weaker compared to the corresponding decline of PM_10_.

**Table 5:** Comparison of models with estimated and observed prevalence of respiratory symptoms and COPD among female never smokers

Time	Exposure	Prevalence [%]		
		Model: PM_10_	Model: NO_2_	observed
Chronic cough				

Baseline	Median exposure ^a^	19.8	20.1	18.9
15 years later	No exposure decline	38.6	28.1	--
	Exposure decline ^b^	26.4	26.4	26.5

Chronic cough with phlegm production				

Baseline	Median exposure ^a^	9.1	8.9	8.8
15 years later	No exposure decline	21.4	14.3	--
	Exposure decline ^b^	13.3	13.0	13.3

Mild COPD ^c^				

Baseline	Median exposure ^a^	8.5	9.4	8.4
15 years later	No exposure decline	39.5	23.1	--
	Exposure decline ^b^	17.5	16.1	18.2

Moderate COPD ^d^				

Baseline	Median exposure ^a^	3.6	4.0	3.2
15 years later	No exposure decline	15.6	8.1	--
	Exposure decline ^b^	6.3	5.6	6.6

^a ^Exposure at baseline (median) of PM_10 _and NO_2_: 48.3 mg/m^3 ^and 46.6 μg/m^3^

^b ^Exposure decline (average) of PM_10 _and NO_2_: 20 μg/m^3 ^and 10 μg/m^3^

^c ^FEV_1 _/FVC < 0.7

^d ^FEV_1 _/FVC < 0.7 and FEV_1 _< 80% reference value

## Discussion

Between 1985 and 2006 air pollution declined with most pronounced changes in industrialized areas as compared to the rural area. In the SALIA cohort we showed that the extent of air pollution decline was associated with a corresponding significant reduction of the age-related increase in prevalence of respiratory symptoms and COPD

Our findings are analogue with the results of the Swiss Cohort Study on Air Pollution and Lung Diseases in Adults (SAPALDIA) study in Switzerland, which observed that decreasing exposure to airborne particles attenuated the decline in lung function in that cohort [29]. Another study of the same cohort also reported a decline in PM_10 _exposure in association with a reduction in respiratory symptoms [30]. In the SAPALDIA study population, whose average age of participants was 41.4 years, the estimated relative decrease of cases with chronic cough for instance that could be attributed to a mean decline of 6.2 μg/m^3 ^ambient PM_10 _over 10 years was 12.2% [30]. Compared to this study, we found a similar relative decrease of the prevalence of chronic respiratory symptoms as well as respiratory diseases in our cohort. The estimated relative decrease of cases with chronic cough for instance that could be attributed to a mean decline of 20 μg/m^3 ^PM_10 _was 31.6%, which correspond to a decrease of 9.8% per decline of 6.2 μg/m^3^, assuming linearity of the association. Other studies investigated the effect of declining air pollution in cross sectional studies: Studies in children from East Germany showed that the improvement of non-allergic respiratory morbidity and lung function in children was associated with declining levels of air pollution [31-33]. Mortality studies showed a reduction in cardiovascular mortality after a decline in ambient air pollution exposure [34]. The previously cited studies and the present one collectively demonstrate a consistent pattern in which reductions in air pollution levels have a beneficial effect on health.

One limitation of our study is our low rate of participation at follow-up relative to baseline for questionnaire items (~50%) and lung function measurements (~15%). Additionally, higher educated women participated more often in the follow-up investigation, therefore, the reported prevalence estimates may be affected by non-responder bias. The main aim of our paper, however, is not to give representative prevalence data, but to estimate whether decline in pollution has a favourable effect on increase of respiratory symptoms and diseases in the elderly. We do not assume that this association may be distorted by non-responder bias since the associations between air pollutants and respiratory symptoms were similar in responders and non responders.

We further assumed in our model that the effect of current smoking was the same in the baseline and in the follow-up investigation (regardless of cigarettes per day or age). In order to address whether potentially erroneous assessing of smoking might have affected the results we additionally did all analysis only including non smoking women into the analysis. The results hardly changed indicating that erroneous assessing of smoking did not bias the effect estimate.

Exposure was characterized by five year concentration means preceding the investigation. Like most other epidemiological studies about effects of long-term air pollution exposure we do not know whether lifetime exposure of the women investigated or current exposure (at the day of investigation) or interactions between chronic and current exposures might modify our results.

The assessment of self-reported symptoms in epidemiological studies is not free from measurement error [35]. For longitudinal studies, specifically, measurement error occurring at both baseline and follow-up may lead to bias of reported incidence estimates [36]. Therefore, we chose to report and model prevalence of respiratory symptoms and diseases rather than the cumulative incidence and remission rates.

The accuracy of self-reported chronic respiratory symptoms and diseases in a questionnaire is difficult for this age group, recall bias may occur and many of the participants may not remember exactly what the doctor had informed them. A study by Medbo *et al. *observed that the reporting of cough, especially with phlegm production, was lower in elderly females than in males, suggesting that cough with phlegm production may not be considered a feminine behaviour [21]. Our study population, however, consisted of women only. We further investigated persons living in urban and rural areas with various levels of exposure. These study locations were chosen to represent a large range of air pollution concentrations. It is possible that elderly women differ in urban and rural areas not only in terms of air pollution exposure, but also in terms of lifestyle and social status factors associated with respiratory health. We included social status as covariate in our analysis. Consistent with previous studies in elderly female populations, we could show that there was no strong association between exposure to air pollutants and socioeconomic status [10,37]. Furthermore, we only observed a marginal and non significant association between respiratory health outcomes and educational level.

Our analysis showed a slightly higher prevalence of mild and moderate COPD in the rural areas compared to women from the urban areas. However, in a previous mortality analysis [10] we could observe a higher air pollution-associated mortality in women from the urban areas, therefore it is possible that women living in urban areas with mild to moderate COPD are already passed away and hence were lost at the follow-up.

We used pre-bronchodilator measurements to define COPD in our study population, however the GOLD criteria recommends post-bronchodilator measurements for the assessment of COPD. We therefore excluded all women who reported asthma at baseline and at the follow-up from our analysis and used a modified version of the GOLD criteria. However, since awareness of asthma has increased during the last 20 years this procedure might have introduced a bias. As a sensitivity analysis we additionally estimated the effects of declining air pollution on COPD without excluding asthma cases. The effect estimates were bigger and the significance stronger. Our results therefore might underestimate the true effect. It is further possible that COPD in older women is overestimated when using FEV_1_/FVC <0.7 for definition. We therefore additionally used moderate COPD (FEV_1_/FVC <0.7 and FEV_1 _< 80% of the predicted) to define 'definite' cases of COPD [38]. This cut off is considered to be more reliable than the GOLD criteria when identifying incidence of COPD in elderly subjects [39]. Irrespective whether FEV_1_/FVC <0.7 in older age reflects a disease, an increase clearly reflects an aging of the lung. Our previous study [6] showed that this lung aging was accelerated in the highly polluted areas at baseline. The results at follow up demonstrate that the increase in lung aging in these areas was attenuated due to a steep decline in air pollution exposure.

To our knowledge, this is the first German cohort study investigating the association between the decline in air pollution and the prevalence of respiratory symptoms and diseases in women followed for more than 20 years. The primary strengths of our study are the long follow-up period of approximately 20 years for these women, and the objective exposure assessment. Furthermore, 98% of the women did not move during the follow-up period; the neighborhood effects for the majority of the participants did not change.

## Conclusion

Parallel to the decline of ambient air pollution over the last 20 years in the Ruhr area a reduction of the prevalence of chronic respiratory diseases and symptoms attributable to air pollutants in a study population of elderly women could be observed. Our findings provide support that the reduction in air pollution appears to attenuate respiratory aging in these women.

## Abbreviations

ATS: American Thoracic Society; BMI: Body mass index; COPD: Chronic obstructive pulmonary disease; ETS: European Thoracic Society; FEV_1_: Forced expiratory volume in 1 second; FVC: Forced vital capacity; GEE: Generalised estimating equations; GOLD: Global Initiative for Chronic Obstructive Lung Disease; NO_2_: Nitrogen dioxide; PM_10 _: Particulate matter with an aero-dynamic diameter less than 10 μm; SALIA: Study on the influence of air pollution on lung function, inflammation and aging; SD: Standard deviation

## Competing interests

None of the authors has any actual or potential conflict of interest including any financial, personal or other relationship with other people or organisations within three years of beginning the submitted work that could inappropriately influence, or be perceived to influence, their work.

## Authors' contributions

TS carried out the follow-up investigation, developed the study design, performed part of the statistical analysis and drafted the manuscript, UR provided feedback to the statistical analysis and helped drafting the manuscript, DS performed the statistical analysis, AV participated in the design of the study and helped to draft the manuscript, TB participated in the design and facilitated the implementation of the study, VH assisted in the follow-up investigation and provided feedback to the draft of the manuscript, UK was coordinator of the baseline and follow-up investigation, participated in the design of the study and helped drafting the paper.

All authors have read and approved the final manuscript.
